# Overcoming doubt: developing CDoH Essentials, a practical tool to introduce the commercial determinants of health

**DOI:** 10.1093/heapro/daae166

**Published:** 2024-11-22

**Authors:** Anna Brook, Katherine Körner, May C I van Schalkwyk, Amy Barnes, Mark Petticrew

**Affiliations:** University of Bath, Claverton Down, Bath, BA2 7AY, UK; University of Sheffield, Regent Court, 30 Regent Street, Sheffield, S1 4DA, UK; Leeds Teaching Hospitals Foundation Trust, St. James’s University Hospital, Beckett Street, Leeds, LS9 7TF, UK; Wakefield Council, Wakefield One, Burton Street, Wakefield, WF1 2EB, UK; Royal Free Hospital, Royal Free Hospital, Pond Street, London, NW3 2QG, UK; London School of Hygiene and Tropical Medicine, 15-17 Tavistock Place, London, WC1H 9SH, UK; University of York, Seebohm Rowntree Building, University of York, Heslington, New York, YO10 5DD, UK; London School of Hygiene and Tropical Medicine, 15-17 Tavistock Place, London, WC1H 9SH, UK

## Abstract

Despite growing awareness of the importance of commercial determinants of health (CDoH), there has been limited development or evaluation of educational and practice-focused support for public health professionals. This article reports findings from an action–research approach bringing together people with academic and practice expertise (*n* = 16) to co-create workshop materials (called ‘CDoH Essentials’), test and improve them through five trial workshops and explore their effects. Five English local public health teams co-facilitated the workshops in their organizations, with participants from public health teams and their internal partners (*n* = 94). Quantitative and qualitative data were collected throughout and analysed to understand: (1) whether the workshops met the expectations of participants, public health and academic observers, and (2) the effects of workshop participation on (a) participants’ knowledge, understanding and critical CDoH literacy, and (b) subsequent working practices and attitudes. The co-created CDoH Essentials appeared effective in meeting expectations, improving knowledge and critical CDoH literacy and promoting action on CDoH. The proportion of participants reporting ‘little’ or ‘no’ CDoH knowledge fell significantly following the workshop (55.4% vs 2.7%). Participants’ increased understanding supported reflection on the implications of the CDoH for their roles and for wider strategy and action. After 3 months, all five settings reported greater consideration of CDoH and had initiated or planned action. CDoH Essentials could be used to galvanize more effective public health action to tackle the CDoH in England and trialled in other public health contexts.

**Contribution to Health Promotion**

- If health and equity are to be prioritized over profit, people in the public health system need to understand what is undermining this goal.
- Our action–research approach produced an intervention (‘CDoH Essentials’) that achieved local aims, met expectations, and increased participants’ knowledge, understanding of their role and confidence in their ability to contribute to addressing CDoH.
- CDoH Essentials could act as Chapter 1 in the public health playbook: a starting point to galvanize more effective public health practice tackling CDoH.
- We propose an adapted framework of CDoH skills and competencies needed for local public health professionals and their partners.

## BACKGROUND

There is growing academic attention on the commercial determinants of health (CDoH) and inequity (De Lacy-Vawdon and Livingstone, 2020; Mialon, 2020; Gilmore *et al*., 2023), mounting recognition of their importance among public health policy and practice communities (Fell, 2022; Pickard, 2022; World Health Organization, 2023; Association of Directors of Public Health UK, 2024) and increasing understanding of the importance of community engagement and leadership in developing effective responses that centre equity and account for the varied experiences of diverse populations (Watson and Martin, 2019; Crocetti *et al*., 2022; Friel, 2023; Sykes *et al*., 2023; Townsend *et al*., 2023; Bohman *et al.*, 2024; Friel *et al*., 2024; Pitt *et al.*, 2024).

Strengthening practitioners’ and decision-makers’ abilities to critically reflect upon commercial factors that determine health and ‘*apply the results of [such] reflection into individual or collective actions for health in any given context’* (Abel and Benkert, 2022)—what we call ‘critical CDoH literacy’—is theorized to be central in implementing comprehensive strategies to address CDoH. The shaping of public understanding and discourse about how health is created and promoted is a well-established mechanism by which policy is influenced commercially. For example, by creating doubt about the certainty of evidence or focusing on personal responsibility for health, corporate actors can affect which, if any, policy solutions are considered or adopted (Madureira Lima and Galea, 2018; De Lacy-Vawdon and Livingstone, 2020; Maani *et al*., 2022; Gilmore *et al*., 2023). Attempting to counter-narratives and propose different conceptualizations of problems and concomitant solutions, whilst working within the existing paradigm, is not expected to be effective; rather, the development of alternative visions and paradigms will be needed (Friel *et al*., 2023).

Knai *et al.* (2018) suggest that interventions targeted at the ‘purpose’ level of a system (i.e. aimed at changing the beliefs and assumptions that shape norms and rules) are difficult to achieve but can be effective. Such interventions require the use of new perspectives and logics to explore what is undermining the creation of a societal system that prioritizes health and equity, and critical reflection on both corporate power and the dominant understanding about how health is created and harmed (Knai *et al*., 2018; van Schalkwyk *et al*., 2022). This indicates the importance of time and suitable methods that enable practitioners and decision-makers, who may have varied beliefs, assumptions and values, to engage in critical reflection and sense-making and to develop confidence and competence to act and advocate in relation to CDoH. Yet, there is limited research on educational and practice-focused interventions aimed at creating the norms, knowledge and critical understanding needed within the public health profession to effectively tackle the CDoH (Freudenberg *et al*., 2021).

It is in this context that an action–research process was implemented in England between March 2023 and January 2024, bringing together people with academic and practice expertise to co-create, test and improve workshop materials (called ‘CDoH Essentials’) aimed at improving practitioners’ CDoH knowledge, confidence and critical CDoH literacy. This is relevant for local action globally, however, acknowledging the work was undertaken in England, we have reflected on contextual matters explicitly to support others considering adaptation to other settings. The intention was to drive change by bridging the gap between CDoH theory and practice.

In this article, we report on the findings of this action–research process, specifically answering the following questions about it:

1. What do different stakeholders (those with relevant academic expertise, public health staff and their internal partners) believe is needed from briefing sessions to contribute to the development of supportive environments for public health action on CDoH at local and regional levels?
2. To what extent can co-created briefing sessions contribute to the development of supportive environments for public health action on CDoH at local and regional levels?
3. What are the pedagogical approaches most likely to be effective in achieving these goals?
4. Are there different approaches that work better for different audiences? (What works, for whom, in what contexts?)

Before describing the methods that inform our findings, we present the context for the study.

### Context for study

Preparatory work included informal scoping conversations about what matters most to policy and practice stakeholders in this field and reviewing research literature recommendations. Stakeholders frequently referenced the importance of cross-organizational support for achieving policy and practice adoption and implementation. They also highlighted that UK public health training does not currently explicitly cover CDoH knowledge or skills. Because much of the action and policy required to improve population health rests in other government departments, public health professionals also need to ensure their colleagues’ understanding and support (Lacy-Nichols *et al*., 2022; WHO Regional Office for Europe, 2024).

The development of briefing sessions for public health teams’ internal partners (such as local authority colleagues including politicians) was identified as an approach that could contribute to supportive environments for public health action to address the CDoH. We conceptualized supportive environments as a range of factors that might enable action to prevent, identify, address or mitigate the harmful effects of the CDoH. Such factors included skills, capabilities, confidence, knowledge, understanding, beliefs and intentions and critical CDoH literacy of people working in local and regional governments, as well as the policies and strategies they have in place.

Public health is often defined as ‘the science and art of preventing disease, prolonging life, and promoting health through the organised efforts of society’ (FPH, 2021). By developing effective practical tools through the involvement of relevant stakeholders in design, testing and revisions, the action–research process aimed to enable public health teams to use skills and evidence to galvanize the ‘organized efforts of society’ towards action on the CDoH.

## METHODS

The action–research approach brought together people with academic and practice expertise to co-create workshop materials, test and improve them through trial workshops in local authorities and evaluate their effects.

### Participants

There were three groups of participants:

1) Academics with relevant expertise (including CDoH, public health policy and practice, knowledge exchange, participatory methods, teaching, inequalities, sociology, health psychology, behavioural science, training and political science);
2) Public Health staff working in local or regional government;
3) Their colleagues (for example, local politicians, those working in economic development, planning, education, communications).

Academics and local public health partners (see criteria for local research sites below) were recruited by the core research team, provided with information leaflets and completed consent forms before taking part in design workshops. Their involvement continued throughout, including contributions to observation, reflection and iteration of the materials. Local public health partners also co-delivered trial workshops in their areas.

Public health partners supported the recruitment of workshop participants based on their local objectives for the workshops. An information leaflet and consent form were circulated to potential participants with the lead researcher’s details for any questions. Further opportunity for consent or opt-out was affirmed at the start of each workshop.

### Local research sites

A convenience sample of five local areas meeting all the inclusion criteria, and willing to participate, was recruited. The inclusion criteria were:

- Local or regional public sector organization (preference for local and regional governments);
- Support from the Director of Public Health or equivalent;
- Public Health practitioner identified as local coordinator and action–research partner;
- At least two internal stakeholders willing to inform the development of content;
- Sufficient participants (6–30) willing to give informed consent to participate in the trial workshop;
- Could attend the design workshops;
- Could provide in-person or online facilities for workshops.

We recruited settings that reflected variation in political control, type of authority (in the UK, different types of local government organizations control different aspects of policy) and urban/rural setting.

### Study design

**Fig. 1:**
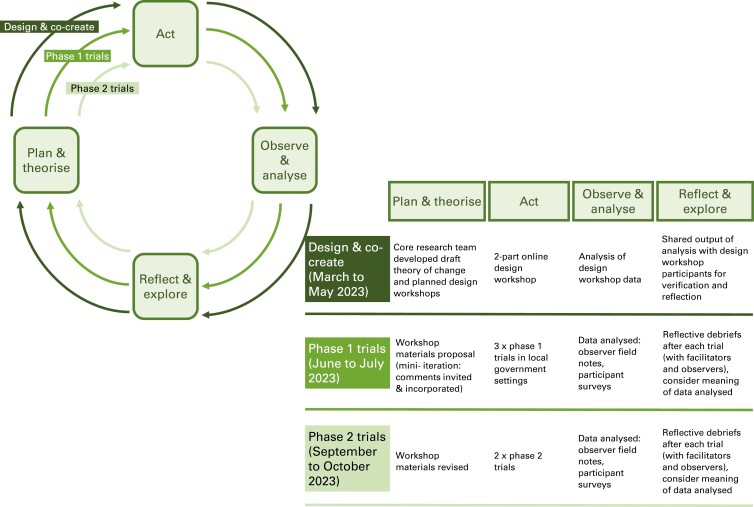
Cycles of action–research, adapted from: Westhorp *et al.* (2016).

Previous research highlights the value of co-creation with both people who will ‘use’ or ‘consume’ an intervention and ‘professional’ stakeholders in yielding original and reliable ideas (Holliday *et al.*, 2015; Leask *et al*., 2019). Therefore, our co-creation processes involved public health partners and participants with relevant academic expertise. The action–research process was conducted iteratively across three main cycles, using a model adapted from that described by Westhorp *et al.* (2016) (Figure 1). This model was chosen to ensure the workshop designs were based on practitioner needs, feedback and contexts.

### Data

The data collected were:

- Written notes, transcripts and outputs (for example, participant-generated online post-it notes and other written content) from two online design workshops.
- Structured written responses to draft and trial materials.
- Surveys of workshop participants pre-, post- and at 3-month follow-up. All consenting attendees were asked to complete a paper survey immediately pre and post the workshop, and we circulated an electronic survey at 3-month follow-up. We worked with action–research partners to attempt to gather feedback. Participants who did not respond were considered lost to follow-up. Surveys included checkbox and rating scales as well as free-text responses. The measures for the participant surveys (and the observation fieldnotes described in more detail below) were based on pre-existing theory about what supports system (Nobles *et al.*, 2021; Bolton *et al*., 2022) and behaviour change (Cane *et al.*, 2012). These theories were chosen because, as explained in the introduction, the beliefs of key individuals within a system are a critical part of dominant paradigms. No existing verified scales measuring the concepts were found. A bespoke survey was therefore designed based on recommendations from the summary of literature on questionnaire design by Lietz (2010).
- Observation fieldnotes and notes from reflective debriefs. Some academic stakeholders and public health partners acted as observers for workshops in different local areas from their own. They took fieldnotes of their observations and participated in a reflective debrief, along with the public health partners from the local area where the workshop was being held and members of the core research team.

Table 1 summarizes the measures and data used to answer each of our research questions.

**Table 1:** Research questions, measures and data

Research question	Summary of measures	Data (brackets show which stakeholder perspectives are covered by these data)
1. What do different stakeholders (those with relevant academic expertise, public health staff and their internal partners) believe is needed from briefing sessions to contribute to the development of supportive environments for public health action on CDoH at local and regional levels?	Expressed and observed preferences for outcomes to achieve in workshops and content and delivery methods used.	Design workshops output (PH and academic) Field notes and reflective debriefs (PH and internal partners) Participant surveys (PH and internal partners)—general comments Q9
2. To what extent can a co-created briefing session contribute to the development of supportive environments for public health action on CDoH at local and regional levels?	Extent to which expectations of participants, public health and academic observers are met Reported changes in knowledge, understanding, beliefs and intentions and critical health literacy (Abel and Benkert 2022), as it pertains to CDoH, of participants Reported subsequent working practices and attitudes	Field notes and reflective debriefs (PH and internal partners) Participant surveys (PH and internal partners) Pre and post: ◦ Self-rated knowledge ◦ Free-text description of ways companies influence health ◦ Free-text things to consider when working partnership ◦ Level of agreement with different statements relating to commercial determinants of health ◦ Skills required (free text) ◦ Self-report on whether have skills required ◦ Level of agreement that ▪ ‘we should be taking action on CDoH’ ▪ ‘it is part of my role’ ◦ Confidence in ability to contribute ◦ Free text on action planned or underway ◦ Aims met ◦ Free text any other comments At 3-month follow-up, we also asked about changes in working practices See Supplementary Materials for surveys and what they were designed to measure
3. What are the pedagogical approaches most likely to be effective in achieving goals? 4. Are there different approaches that work better for different audiences? (What works, for whom, in what contexts?)	Expressed and observed engagement with content and delivery methods used, any indications of which outcomes are supported, any differences in engagement by type of participant or setting	Design workshops output (PH and academic) Field notes and reflective debriefs (PH and internal partners) Participant surveys (PH and internal partners)—section 3 gave some contextual information

To explore what different stakeholders believed was needed from workshops, we used a range of methods. Data from design workshops and responses to draft and trial materials allowed us to understand what public health partners and those with relevant academic expertise expressed as their preferences. Our observations about public health partners’ choices in designing and delivering their local workshops also enabled us to make judgements about their implicit preferences. The fieldnotes from observations and reflective debriefs gave us insight into the expressed and observed preferences of different stakeholders. The post-workshop survey free-text responses also contributed to this question.

To explore to what extent a co-created briefing session could contribute to the development of supportive environments for public health action on CDoH at local and regional levels, we used reflective debriefing and observation fieldnotes and a range of responses from the pre-, post-, and 3-month follow-up participant surveys. We set out three broad areas as indicators of whether the workshops were contributing to the development of supportive environments:

- Extent to which expectations of participants, public health and academic observers are met;
- Reported changes in knowledge, understanding, beliefs and intentions and critical CDoH literacy of participants;
- Reported subsequent working practices and attitudes.

The data collected were designed to identify these three indicators.

We reviewed data from design workshops, reflective debrief and observation fieldnotes, and participant surveys for insight into expressed and observed engagement with different methods used, and any contextual differences in engagement.

### Analysis

As outlined above, this study had two distinct but interlinked aims: to co-create workshops and to evaluate their effects. Our focus in analysing the data was to produce a broad response to the research questions and to identify learning points to inform the development of a practical tool, rather than a full and rich analysis of participants’ perspectives or the meanings that may be derived from analysing their spoken contributions. This intention is reflected in our reporting of the data.

In approaching the analysis, we were aware of our own perspectives as public health scholars with an interest in changing the current systems that perpetuate inequalities and in ensuring greater action to address the CDoH. We viewed our different expertise within the research team as a strength: yielding insights to incorporate into the work from wider CDoH and public health academic literature and from direct local public health practice experience. However, we share this information in recognition of the importance of reflecting on how our perspectives and experiences inform our interpretation and presentation of our findings. The value we placed on co-creation and reflective practice is also apparent in our approach to data collection and analysis. We collected data that allowed us insight into a range of stakeholder perspectives including reflective discussions. We acknowledge that our position as the core research team who have chosen the words that go into this article means we will inevitably privilege our interpretation, however, we made several attempts to ensure the meanings assigned by the participants were valued and amplified within our write-up. For example, by checking our interpretation of data with academic and public health partners and remaining attentive to our own values when discussing interpretation and themes as a group.

Although we reflected as a team on our learning throughout the action–research process, we also analysed qualitative data at the start of the process using framework analysis (Gale *et al*., 2013). The lead researcher undertook familiarization (reading through all data and listening to the recordings of the design workshops to check notes) and open coding (also noting key ‘takeaways’ and impressions). Initial frameworks were developed from a combination of the research questions and the outcomes sought and these were compared with the open codes from the data to adapt the frameworks. The lead researcher applied the frameworks to the data using tables. A series of tables were then used to chart the data by theme (framework matrix). This process was both deductive and inductive with new categories and domains created, guided by reflexive analysis of the data. A second researcher separately reviewed the coding, application of the frameworks and charting. Discussions between these researchers at each stage were used to reflect on the interpretation of data, and its allocation to one or more domains, add to codes and adapt frameworks.

Data were combined at the interpretation stage and considered together under each of the research questions (O’Cathain *et al.*, 2010). This involved reviewing all the data that contributed to each research question and reflecting, as a team, on areas where different data converged, diverged or added new meanings or depth to our understanding. The creation and development of themes was done through reflective writing, to explore each theme fully, along with dialogue between the core research team, to support this analysis process (Ralston *et al*., 2024).

In each cycle of the action–research, a summary of the initial interpretation and themes was presented to academic and public health partners for comment or amendment. In the design phase, a quick turnaround between the two-part design workshops meant the second workshop could reflect and build on themes from the first. At the end of the project, in addition to circulating the analysis and draft practical tool for comment, two drop-in sessions were held for partners to discuss the emerging findings and their implications. These reflective discussions were important in bringing together different types of knowledge, understanding and expertise to create shared understandings and identify areas of difference.

### Ethics

The research received ethics approval from the London School of Hygiene & Tropical Medicine (ref: 28427).

## RESULTS

### Trial workshops

All workshops were held in-person and workshop length ranged from 90 min to half a day (Table 2).

**Table 2:** Local research trials descriptive data

		Phase 1 (*n*)	Phase 2 (*n*)	Total (*n*)
Regions	East Midlands		1	1
	North-East	1		1
	Yorkshire and Humber	2	1	3
Political control	Conservative		1	1
	Labour	3		
	LibDem		1	1
Type of authority	Mix of metropolitan, upper tier/county councils and unitary authorities Mix of urban and rural areas			
Mix of participants by phase	Mainly public health (including wider teams such as research, community safety, community wellness and leisure, libraries, environmental health)	2	1	3
	Mixed participants: both public health and their internal partners (including councillor, economic development, communications, trading standards, planning, climate change, children’s, health and safety)	1	1	2
Participants	Invited			135
	Accepted			106
	Attended			94
Surveys	Pre-workshop surveys completed			86
	Post-workshop surveys completed			75
	Follow-up surveys (at 3 months) completed			39

NOTE: In making pre- and post-comparisons of quantitative data, we have excluded workshop 1 for which we were unable to collect post-workshop data.

### Findings

**Fig. 2:**
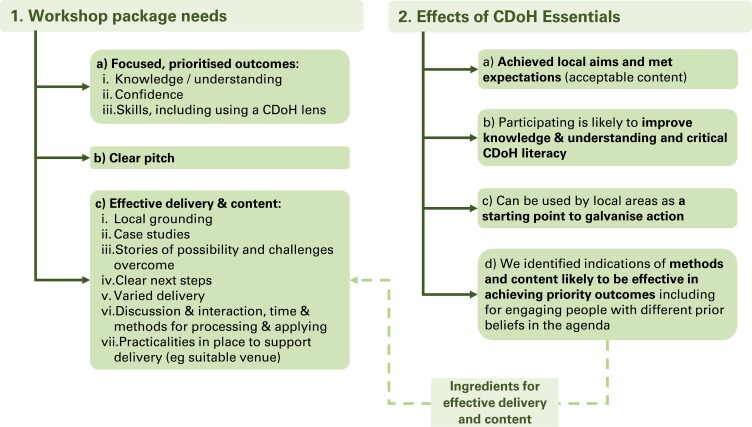
Summarized themes.

Figure 2 summarizes all the themes and sub-themes we felt were important for practitioners wishing to make use of the practical tool, CDoH Essentials. However, for the purposes of writing this article, we have chosen to present selected findings under two main headings. These are the findings that we felt would benefit from further detailed description to illuminate the nuances of the concepts explored.

1. Workshop package needs

Stakeholders believed a range of elements were needed for CDoH workshops:

a. A set of **focused, prioritized outcomes**: priority outcomes were improved knowledge/understanding, confidence and skills, including using a CDoH lens.
b. A **clear pitch:** to encourage attendance, *‘something quite black and white about what they’re coming to and why’ (local public health partner, design workshop)*, as well as to explain how introductory sessions fit with local strategic objectives for CDoH and likely next steps.
c. **Effective content and delivery.**

We identified seven different aspects relating to effective content and delivery of sessions (i.e. things that effective workshops should have). These seven aspects were produced collectively between the core research team (who reviewed data in depth) and academic and public health partners (through shared reflective discussions to revise and create the final list).

i. Local grounding
ii. Case studies
iii. Stories of possibility and challenges overcome
iv. Clear next steps
v. Varied delivery
vi. Discussion and interaction, time and methods for processing and applying
vii. Practicalities in place to support delivery (e.g. suitable venue).

i) Local grounding

‘Local grounding’ involves sessions being ‘made personal to their context within their communities’ (local public health action–research partner and academic participant, design workshop 1). At the design stages, it was considered important to ground the workshops in the local context and to connect content with issues of relevance to participants’ work and remit (including inequalities and connections to the social determinants of health), as well as to explain how systems affect individual choices.

The importance of locally relevant material was reinforced in debriefs, observation fieldnotes and participant surveys. For example, when material was not sufficiently locally relevant, this was raised as a specific debriefing discussion point:

Didn’t link back to impact on residents in a really local and obvious way – perhaps gambling would do this more effectively, perhaps need to bring in local stats and facts to do this too. (Workshop 1 debrief)

It was also observed that participants demonstrated engagement (i.e. nods, note-taking) when this was done well:

Concise, clear, related content back to the previous local-level contextual presentations. Example of food choices – lots of nods, people taking notes. (Workshop 5 observer fieldnotes—food slide connecting wider determinants of health framework with CDoH)

iii) Stories of possibility and challenges overcome

There was some discussion about the importance of including stories of possibility and challenges overcome in the design stage, which we initially conflated with ‘next steps’ and ‘case studies’. However, we noted that there were distinct data and conceptualizations for each of these ideas, so we chose to separate them to ensure that important meanings were not lost. Again, the importance of this aspect of effectiveness was highlighted both from supportive comments when this was done well and as an area for improvement when missing or insufficient. For participants, it meant knowing that: ‘people like us have done this, and it’s made a difference’ (academic participant, design workshop 1).

vi) Discussion and interaction, time and methods for processing and applying

Having time for discussion and interaction, as well as time and methods for processing and applying learning, was not explicitly discussed by partners in the design stages. However, the importance of these elements became apparent as we reflected on observer fieldnotes, in debriefs and participant feedback: as a team, we felt it was implicit in the types of activities that were suggested and chosen for the trial-workshop agendas by local research partners. Discussions were important in how learning progressed within workshop sessions: ‘discussions I observed were fruitful and relevant, people spinning off each other with more and more examples’ (workshop 5 observer fieldnotes ref evidence quest). ‘Methods’ as well as ‘time’ both seemed important to participants for processing knowledge and applying understanding:

The feedback from groups suggested that people had reflected on what implications the case study might have based on own role/context. (Workshop 3 observer fieldnotes ref case study)

When this was missing or insufficient, it was noted as a gap/area for improvement.

vii) Practicalities in place to support delivery

Reflective debriefs, fieldnotes and participant feedback all indicated that several ‘basics’ need to be in place—time, pace, flow, balance of activities, room layout, comfort, refreshments, breaks—for people to engage. When analysing our data, we reflected on what this might mean for CDoH specifically. These are things that would be relevant for any workshop, so we discussed as a core research team and with academic and public health partners what might be particularly important for practitioners wanting to run CDoH workshops.

We had received a few comments suggesting that there was a lot of information to digest. These were exclusively from workshop 3 (in the first phase) where the timing and room layout were most problematic. Second phase areas learned from earlier workshops meaning these areas were more likely to have the basics in place. We felt this suggested that the practicalities of the session may affect people’s feelings about CDoH and that, when the basics were not in place, this could negatively affect participants’ ability to reflect on CDoH and begin to apply this reflection to action:

People needed time to process and they didn’t really get it – refreshments and break might have been good. (Workshop 3 observer fieldnotes)

2. Effects of CDoH Essentials

We found that CDoH Essentials:

a. Achieved local aims and met expectations;
b. Was likely to improve knowledge, understanding and critical CDoH literacy;
c. Can be used as a starting point to galvanize action;
d. Comprised content and delivery methods that seem effective in achieving priority outcomes, including for engaging people with different prior beliefs in the agenda.
a) Content was acceptable, achieving local aims and meeting expectations

Based on our analysis of the quantitative data, we found that the action–research process resulted in a workshop that met participants’ expectations. Overall, 88% of participants (at least 75% in each workshop) reported the aims were met well or very well (95% CI 80–95%). We also reflected on how the qualitative data supported this idea or whether there were any amendments to make. The reflective debriefs from most workshops highlighted the successful engagement of participants:

People were engaged – writing notes, nodding, listening, asking questions, the observers noted that no-one was looking at their phones and switching off. (Workshop 1 debrief)

Good conversations – better than might have thought. (Workshop 4 debrief)

Comments on workshop 3 were slightly more varied, including some more qualified comments mixed with some more positive ones:

Went ok people seemed engaged, subject matter appealing and relevant…pretty good…Nice level of engagement…Felt like good energy in the room everyone contributing. (Workshop 3 debrief)

Participant feedback post-workshop and at 3-month follow-up was overwhelmingly positive (e.g. *‘excellent’, ‘enjoyable’, ‘useful’, ‘very interesting’, ‘thought-provoking’)* with some suggestions for improvement and only a few mixed or negative responses. These are related to practicalities and have been considered as part of the discussion on the importance of getting the basics right under theme c.vii above.

There were several comments in the post-workshop survey about wanting longer/more. Some were positively framed:

loved it - could have spent longer as it was so engaging and time went quickly. (Workshop 2 participant survey response)

Some were negatively framed, exclusively from one workshop in which the timing was particularly restricted:

Needed longer - felt quite rushed. (Workshop 3 participant survey response)

b) It is likely participating in such a workshop improves knowledge, understanding and critical CDoH literacy

Bringing together quantitative and qualitative data and reflecting on where the workshops appeared to be most successful, we found that participating in the workshops appeared to improve knowledge, understanding and critical CDoH literacy:

i. Knowledge and understanding

The quantitative data from the surveys were, as anticipated, useful in providing a quick descriptive summary of changes from before to after the workshops:

Those reporting CDoH knowledge as ‘little’ or ‘none’ fell from 55.4% to 2.7% following the workshop (95% CI 40.1-64.6)

Our analysis of participants’ free-text responses then enabled us to consider the different dimensions of knowledge and whether there were indications that understanding was deepening in any areas. For example, when asked to describe the CDoH, we noted an increase in the breadth and depth of responses after the workshops. Responses before the workshop were more likely to list specific products or industries without describing how they affected health, or to list just one type of commercial practice (often some form of advertising/marketing) whereas, after the workshops, more responses listed a range of practices.

Where there was already a high level of prior understanding, we did not see such substantial changes in the quantitative data. It is likely that participating in the workshop maintains or increases prior high levels of certainty in the evidence. Post-workshop, almost all participants agreed or strongly agreed with evidence statements (see Supplementary Materials for figures). The effect was larger for policy influence, where the prior agreement was lower. This was similar in free-text responses: when asked to list key considerations before starting a partnership, there was a good breadth of responses both before and after workshops.

We then considered observation fieldnotes and debrief data. There were several comments that indicated the material seemed to work well for increasing knowledge and understanding:

Overall the material landed well, people engaged with it – demonstrated by no incongruous or off topic questions or discussions, no one said anything that implied they weren’t following the thrust of the workshop, even if they disagreed. (Workshop 1 debrief)

ii) Critical health literacy

Again, the quantitative survey data from participants gave us a quick indication of improvements achieved.

24.7 percentage-point increase in those agreeing or strongly agreeing that it is part of their role to take action on CDoH (95% CI 11.3 to 38), from 63% pre-workshop.

27.8 percentage-point increase in those agreeing or strongly agreeing that they are confident they can contribute to action on CDoH (95% CI 12.5 to 43), from 45.2% pre-workshop.

21 percentage-point increase in those agreeing or strongly agreeing they have the skills they need to contribute to taking action on the CDoH (95% CI 5.1 to 37), from 38.3% pre-workshop.

In our initial impressions from the reflective debriefs and observation fieldnotes, we also identified that participants had started to make connections to their own roles and practice critical thinking and other skills. An example of the data we highlighted in support of this impression is:

The feedback from groups suggested that people had reflected on what implications the case study might have based on own role/context…suggesting that they were relating to materials to their own role/knowledge. (Workshop 3 observer fieldnotes ref case study)

We brought these two sets of data together, along with some of the participant free-text survey responses, and discussed the different aspects of the theme as a core research team. We noted that it made sense to combine the different elements into a single sub-theme on critical CDoH literacy. We then went back to review the data with this in mind and brought in some of the comments relating to how participants processed the knowledge obtained, built their understanding, reflected on how to apply it to their role, and what action to take:

Didn’t feel totally overwhelming – felt like we helped people engage with a complex issue and that they felt they could do something as a next step. (Workshop 1 debrief)

As described in other themes, some participants commented on the need for more time to process and digest information. Therefore, in constructing this finding, we felt we needed to emphasize that these were introductory workshops, and the data reflect that.

Participants were able to describe some of the skills they believed they needed to address CDoH. We have used their responses to propose (in Table 3) an adapted framework of skills and competencies needed for public health professionals and their partners to address the CDoH at the local level, which builds on that suggested by Freudenberg *et al.* (2021).

**Table 3:** Proposed framework of skills and competencies for local and regional public health system partners

Skills, competencies and characteristics required for people working on CDoH in local government		Connections to the Freudenberg *et al.* (2021) framework
Define CDoH Recognize examples of how CD affect health Explain CDoH in relation to other PH frameworks	Ability to define CDoH, and recognize examples of the different practices and processes, analyse and describe how practices and processes affect health and explain relationship to other widely used Public Health frameworks such as wider/social determinants of health.	Define CDoH and discuss its history and evolving conceptions of its meaning, importance and relationship to other determinants (e.g. biological and behavioural) and public health frameworks such as social determinants of health.
		Assess marketing practices and corporate political activity among major health-harming industries such as tobacco, alcohol, food and beverage, pharmaceuticals, social media, fossil fuels and others.
Use a CDoH lens for work (both analysis and development of research, practice and policy)	Ability to use a CDoH ‘lens’ for own work, a) applying relevant frameworks to the analysis of public health practice, research and policy analysis b) to be able to develop research studies and interventions (including engagement with the public and other appropriate stakeholders) that contribute to effective strategies for minimizing the harms and maximizing the benefits of CDoH.	Apply CDoH frameworks to the analysis of public health practice, research and policy analysis to be able to develop research studies and interventions that contribute to effective strategies for minimizing the harms and maximizing the benefits of CDoH.
Identify key sources of evidence and data	Identify key sources of evidence and data on the distribution, impact and pathways by which CDoH influence health and inequalities and assess the strengths and limitations of these sources in order to apply effectively in practice.	Identify key sources of evidence and data on the distribution, impact and pathways by which CDoH influence health and assess the strengths and limitations of these sources.
Critical thinking and questions	Ability to critique evidence, and its sources, from a CDoH perspective, taking account of potential commercial, political and other biases	
CDoH communication	Strong communication skills that reflect the CDoH evidence base (e.g. norm shaping).	Make the case for public health practice and research that address CDoH as fundamental determinants of health and health equity.
Community-focused practice	Community-focused practice, including: understanding of local population, engaging and advocating with communities, developing and building trust and effective methods for exposing communities to CDoH evidence.	
Advocacy and influencing	Advocacy and influencing skills to improve public health and increase equity (esp. re-policy and decision- makers) including skills to work in political environment such as negotiation and leadership	
Make case for CDoH informed practice	Make the case for public health practice and research that address CDoH as fundamental determinants of health and health equity.	
Assess public health CDoH strategy for impact	Assess the various strategies, tactics, counter-marketing and campaigns by advocacy groups and coalitions to address the harms of CDoH and help reduce noncommunicable diseases and other adverse outcomes. Assess the strengths and weakness of various supply-side and demand-side government policy solutions and intergovernmental agreements to reduce noncommunicable and other diseases to enable rigorous support or critique of national policy	Assess the various strategies, tactics, counter-marketing and campaigns by advocacy groups and coalitions to address the harms of CDoH and help reduce noncommunicable diseases and other adverse outcomes.
		Assess the strengths and weakness of various supply-side and demand-side government policy solutions and intergovernmental agreements to reduce noncommunicable and other diseases.
Understand possibilities for action at LA level	Understand possibilities for action at LA level—frameworks and ‘best buys’ evidence (with worked examples from practice)	
Leadership for public health advocacy	Leadership skills including building relationships and networks for public health advocacy	
Confidence, resilience and persistence	Confidence to act (as collectives, with support from others, based on better understanding of CDoH evidence and improved skills), resilience (understood to be achieved through supportive networks) and persistence (defined mainly as willingness to persevere despite setbacks).

c) Areas that run such workshops can use them as a starting point to galvanize action

Although action is part of our conceptualization of critical CDoH literacy, we chose to draw this aspect out as a distinct theme because of its significance to us and to our local research partners. Our understanding of ‘action’ encompasses changes in ways of thinking and approaching work, as well as more tangible activities such as the adoption of a policy or running a training session.

The quantitative data from participant surveys gave us a strong starting point, indicating that it is likely workshop participation maintained or slightly increased existing high levels of agreement that we should be taking action on the CDoH

8.3 percentage-point increase in those agreeing or strongly agreeing (95% CI 0.19 to 16.3), from 89% pre-workshop.

Participants described a wide range of actions when asked about what they had planned or underway, both in pre- and post-workshop surveys and within planning activities during the trial workshops. To give us a sense of the breadth of understanding of possible actions, we coded responses against the frameworks introduced during the workshops (Friel *et al*., 2023; Jawad and Reed, 2023). Responses covered all aspects of the frameworks (apart from price as a mechanism for reduced sale and consumption). The main addition to the existing frameworks was alliance building (within which we also captured: extending training to others, community-focused practice and awareness raising—which also connects with the communication theme).

As outlined above, design workshop participants agreed it was important to set out *‘the solid next step’ (local action–research partner research site B, design workshop 1),* and this was supported by fieldnotes, debriefs and participant feedback. Some felt more time should have been given to this in the sessions, others felt a break before applying knowledge and understanding would be better. There was one comment that mentioned being unclear about what to do next.

From the quantitative data, it was unclear whether workshop participation affected optimism that acting on CDoH would result in positive outcomes or whether it was context-dependent. In two groups there was no/neutral impact; in one optimism decreased, and in another, it increased. We, as a core research group, discussed this with partners and reflected that optimism may be affected by the substantial struggle that confronts local areas in taking on powerful actors and systems, but also that we could recommend trying further improvements, addressing this point, to local areas using the materials. We did not specifically ask about feelings towards action in questions with free-text responses, but, in reviewing the data, we looked for any spontaneous mentions, which we felt would give us further insight into this issue. Most comments relating to feelings about action were positive, e.g.

action on CDoH feels more attainable and achievable. (Workshop 5 participant survey response)

After 3 months, individual-level reports on changes that participants had made following the workshop were mixed. However, when aggregated to the area level, all five settings reported some increased awareness, discussion and/or consideration of CDoH, and some form of specific action started or planned.

Overall then, the naming of this theme reflects that local areas were positive about how workshops created opportunities for action and affected its planning and instigation, participants were mostly positively oriented towards action, and some early indications of action and change were seen in each area, but it was just the start.

d) We identified indications of methods and content likely to be effective, including for engaging people with different prior beliefs in the agenda

In constructing this theme, we reflected that there was a potential overlap between what academic and local authority partners believed to be important and what we found was likely to be effective. We have chosen to report the domains of effective delivery and content in workshop package needs, theme c above. However, in our analysis of observation fieldnotes and participant data, we also noted indications that all seven aspects are likely to be effective in achieving priority outcomes, as well as being believed to be important. We have illustrated this with a few areas where the data seemed to us to offer some additional insight.

For example, the introductory presentation is likely to be important for baseline knowledge, with a couple of sections highlighted strongly. First, the use of a known example (tobacco control) to introduce the Lancet framework:

Lots of nods when used tobacco examples. Known example aided understanding of theory. (Workshop 3 observer fieldnotes)

Second, the slide illustrating structural and systemic issues using wider determinants of health framework and layering on the CDoH, illustrated with the food system. The identification of specific aspects of content and delivery that seemed to contribute to specific outcomes was important for developing recommendations for the practical tool.

Similarly, we highlighted above that it was believed that ‘stories of possibilities and challenges overcome’ would be an important element for enhancing effectiveness. We reflected that that this did seem to be an important aspect in practice, based on data such as fieldnotes observing higher energy in the room when discussing actions, and participant feedback requesting even more examples of actions that could be taken.

Public health research partners designed their agendas based on what they anticipated would work for their attendee mix. We feel this approach of using local understanding to design workshops seems to have been effective because both types of attendee mix reported positive feedback.

In reflecting on what seemed to work for engaging those with differing prior beliefs, debriefs and fieldnotes suggested specific aspects of presentation, evidence quest and case studies were all important.

Deliberately focussing on harms, while being aware there might be positives. Important to mention this, given that the audience may be diverse in political beliefs/level of CDoH understanding. (Workshop 3 observer fieldnotes)

Where there was an expressed view that individual choices were more important than structural, and some discussion about this in the workshop, the debrief afterwards suggested the tone and approach of facilitators were important and that, since the individual offered to be involved in subsequent activity, they were not put off.

Not helpful to challenge too directly, need to listen – feeling this went well. (Workshop 1 debrief)

## DISCUSSION

We reflect here on the main results in relation to key research questions, and what they imply for future action on CDoH.

1. What do different stakeholders (those with relevant academic expertise, public health staff and their internal partners) believe is needed from briefing sessions to contribute to the development of supportive environments for public health action on CDoH at local and regional levels?

Stakeholders across the three groups brought different perspectives but there was a high level of agreement about workshop package needs, especially the aspects of content and delivery considered to be important in achieving aims.

These insights have been used to guide the current form of the practical outputs (Brook *et al*., 2024). They would benefit from further testing to explore whether they are effective in different settings (including different countries), for different audiences, and to achieve different outcomes.

2. To what extent can a co-created briefing session contribute to the development of supportive environments for public health action on CDoH at local and regional levels?

The approach was found to be feasible and adaptable. It is likely participating in workshops designed using our materials will improve participants’ knowledge, understanding and critical CDoH literacy. The workshops can be used as a starting point to galvanize local action on CDoH. Further research with longer follow-up could review whether the reflection, and beginnings of action observed and reported, develop into further individual or collective action and lasting changes in beliefs and practice.

It would be useful to explore further whether the roll-out of such sessions to wider groups does, over time, contribute to the development of supportive environments for public health action on CDoH. Recognizing that no single intervention can be optimally effective in isolation, it would be informative to determine the range of interventions needed as part of a more comprehensive strategy to prevent and counter the harmful effects of the CDoH at local and regional levels. Learning networks that facilitate the dissemination of local knowledge and experiences were suggested by public health research partners as a starting point.

3 and 4 What are the pedagogical approaches most likely to be effective in achieving these goals? and Are there different approaches that work better for different audiences? (What works, for whom, in what contexts?)

Most participants already believed improving public health to be important and most already believed structural approaches were important. We know that public perceptions about what creates health tend to be more focused on individual factors than the wider determinants of health (L’Hôte *et al*., 2018) and we explored participants’ mental models about health before and after the delivery of the briefing session using a set of questions developed from the critical health literacy scale (Chinn and McCarthy, 2013), the Dahlgren and Whitehead social determinants of health model (Dahlgren, 1993) and the Health Foundation and Frameworks Institute work on the social determinants of health (L’Hôte *et al*., 2018; Elwell-Sutton *et al*., 2019). This contextual information indicates that our findings may not be applicable to those who believe population health is mainly the responsibility of individuals or who believe, at either extreme, that it is entirely the responsibility of the government or entirely the responsibility of individuals. Although we have indicated that some content and delivery seemed to be effective for engaging people with differing prior beliefs in the agenda, further research to explore engagement with these materials by groups with greater differences in beliefs will be important.

### Fit with existing literature

The development of CDoH Essentials responds to calls from the CDoH literature (and draws upon it) to produce practical tools to help public health practitioners and others take effective action (Freudenberg *et al*., 2021; Lacy-Nichols *et al*., 2022; Friel *et al*., 2023; WHO Regional Office for Europe, 2024). The aims, messages and design are likely to be of general relevance, although they will require adaptation and extension for other contexts, to reflect local challenges and levels of resources, particularly in the Global South where the industries and commercial practices of most concern, and the concomitant public health challenges, may be different. Many aspects will be transferrable—particularly given the highly consistent nature of practices adopted across industries (Ulucanlar *et al*., 2023) and we have reflected below on transferability considerations.

CDoH present perennial and evolving challenges (Knai *et al*., 2018; Friel *et al*., 2023; Gilmore *et al*., 2023). This means there is a need to consider how institutional memory and learning around CDoH is maintained and built on. Our model of education aims to support this by going beyond simply ‘transmitting’ information, towards enabling what we termed critical CDoH literacy, adapting Abel and Benkert’s (2022) definition for our purpose. For us then, this meant: helping others to develop new ways of conceptualizing public health problems and their causes, drawing on different disciplines and theories and giving people enough time and space to reflect on and consolidate their learning and be activated by it. CDoH action is not simply the prerogative of practitioners and academics and much effective action has been taken by NGOs, communities and the wider public (including young people); weaving their experience and expertise into such training will contribute valuable insights, help build trust and trustworthiness and add credibility (WHO Regional Office for Europe, 2024). Public health professionals have a role to play in building wider understanding of the CDoH among the public, which could draw on developments in areas such as climate communications (van der Linden *et al*., 2017; Farrell *et al.*, 2019), framing (Katikireddi *et al.*, 2014; Rowbotham *et al*., 2019; Maani *et al*., 2022) and other rhetorical practices and storytelling (Lillie *et al*., 2024). These will require ongoing education and skills development to stay abreast of industry strategies and ensure communications and other actions are an effective counter to these.

## STRENGTHS AND LIMITATIONS

### Strengths

The action–research methods resulted in a toolkit that is acceptable to all stakeholders (academics with different expertise, local public health and their internal partners). The iteration allowed us to learn quickly and improve throughout the process. The involvement of different stakeholders meant careful attention was given to different aspects of what might be important—ensuring the CDoH evidence base was reflected carefully in the materials, ensuring lessons from education and behavioural psychology were considered in the design, ensuring the materials were suited to different participants and ensuring the toolkit and guidance were useable by public health teams without the support of a research team. Time spent thinking about why and how things might work (upfront theory), and then reflecting about what was observed and specific contextual factors enabled us to attempt to go beyond a simple transmission of information and begin to consider what might help change ways of thinking.

### Limitations

This is an initial scoping study that involved a limited range of stakeholders and settings. Additionally, we were unable to collect data post-workshop 1 and, because this was one of the two mixed participant groups, this limited our ability to explore differences between public-health-only and mixed groups. The sustainability of effects is also unclear; there was a high loss to follow-up at 3 months and we did not assess whether changes in knowledge, understanding and other effects were maintained.

## IMPLICATIONS AND RECOMMENDATIONS

Whilst public health teams found CDoH Essentials useful for themselves, they were also helpful in their intended purpose of engaging wider partners. We encourage colleagues to go beyond the public health team and make use of the toolkit with their communities to support change through the ‘organized efforts of society’.

We recognize that every place is different: the CDoH challenges, and the resources to address them, will vary widely between countries and settings, as will the priorities of stakeholders and the public in confronting these challenges. The CDoH Essentials provide a starting point, but they will need adaptation to meet local needs and how these change with time. To support this, we have reflected explicitly (both in this article and the toolkit) on contextual issues in the trial areas that may affect transferability and we have developed a set of transferability considerations (see Table 4) to share what we have learned with other teams who may wish to adapt these for their contexts (including other country settings). Similarly, we encourage users in other settings to evaluate and report on their experiences of the adaptation, use and impact of the CDoH Essentials, and call on global bodies such as WHO to facilitate the sharing of learning.

**Table 4:** Transferability considerations

	Considerations include
Purpose	Current overall purpose, principles, values and goals in your context—both as they are explicitly described and implicitly enacted (and any differences) Your desired overall purpose, principles, values and goals (and how these may differ to current context) Your long-term goals for work on CDoH, how these fits with and differ from your desired purpose, principles and goals and the current context, and how the briefing session/s may contribute to any desired changes
Actors/stakeholders	Implicit and explicit beliefs and norms in your context and of target audience—for example, understanding and expressed beliefs about how health is shaped, for example, whether there is an accepted view about inherent benefits from working in partnership The needs and goals of target audience/s—both for long-term work on CDoH and specifically for any briefing session/s planned
Context	Analysis of existing policy, strategy, resources, power: identification of those elements that are operating in alignment with or against your desired overall purpose, principles, values and goals—you could use the model of the CDoH from the Lancet series: figure 1, paper 1 (Gilmore *et al*., 2023) to review the different elements of the system for your context Community/population assets and needs You and your team—what is your style? What are your strengths, weaknesses, resources? Analysis of the above to understand: where are the opportunities? Where are the risks? What are the ‘hot topics’ that are important right now and will engage people? How is this relevant to what matters to you (whole population and community) right now?
Dynamic situational analysis	All of the considerations listed above are likely to change and respond to changes you make. How will you ensure that you can stay up to date and scan the horizon for emerging issues as commercial practices change, as local assets and needs change, as actors and stakeholders change?

## CONCLUSIONS

This action–research project yielded insights about what is likely to work (or not) in different contexts and resulted in the development of the CDoH Essentials toolkit which met its aims, including increasing participants’ critical CDoH literacy. We have also proposed a framework for skills and competencies built on the suggestions from Freudenberg *et al.* (2021), which would benefit from substantial further development with broader audiences through a bespoke approach. A strong focus on contextualizing the findings should mean that practitioners in different settings can consider whether the insights are applicable or adaptable to their situation.

Finally, this is really only Chapter 1 in the public health playbook—more awareness raising, alliance building, action development and other work is needed. Public Health teams can use and adapt the materials to generate understanding and start a plan of informed action based on critical reflection on the current systems that affect our health. CDoH Essentials provides a strong starting point for local teams to galvanize action to prioritize health and equity and address the CDoH.

## Supplementary Material

daae166_suppl_Supplementary_Material

## Data Availability

The data that support the findings of this study are available on reasonable request from the corresponding author. The data are not publicly available due to privacy or ethical restrictions.
