# Supplementary material for: Overcoming doubt: developing CDoH Essentials, a practical tool to introduce the commercial determinants of health
Source: Health Promot Int. 2024 Nov 22;39(6):daae166. doi: 10.1093/heapro/daae166 (PMC11582073; doi:10.1093/heapro/daae166)
Supplement: daae166_suppl_Supplementary_Material [file daae166_suppl_supplementary_material.docx]

**Supplementary material**

There are four sets of supplementary materials for publication online

# Supplementary material 1: Outcomes

## Outcome dimensions and measurement

| **Outcome dimensions** | **Measurement approaches** | **Explanation** |
| --- | --- | --- |
| Understanding: what level of understanding do people have about the commercial determinants of health? | Pre- and post- survey question 1: self-rated knowledge | We wished to know about self-rated knowledge before and after the delivery of the briefing session. We aimed to improve knowledge. |
|  | Pre- and post- survey question 2: ways companies influence health | We wished to know what participants understand about how companies influence health before and after the delivery of the briefing session and whether this has changed. |
|  | Pre- and post- survey question 5: things to consider when working in partnership | This is an open question which some may not feel able to answer. We have not given examples because we do not wish to lead participants. We want to understand what comes to immediately to mind when they think about working in partnership (both before and after the briefing session). |
|  | Observation and field notes | To note whether there were observable differences in understanding during the workshop and whether this was associated with any particular content or delivery methods |
| Beliefs: what are people’s beliefs about the importance of this agenda (vs other agendas?) | Pre- and post- survey question 3: | We know that public perceptions about what creates health tends to be more focused on individual factors than the wider determinants of health. We wish to understand participants’ mental models about health before and after the delivery of the briefing session. This is expected to provide context that may help us to understand whether some of our approaches work better for some than others. The options in the survey were developed from the critical health literacy scale (Chinn and McCarthy 2013), the Dahlgren and Whitehead social determinants of health model (Dahlgren and Whitehead 1993) and the Health Foundation & Frameworks Institute work on the social determinants of health (L’Hôte et al. 2018; Elwell-Sutton T et al. 2019). |
|  | Observation and field notes | To note whether any expressed differences in beliefs affected observable engagement with the materials |
| To what extent do people believe any change is needed? | Observation and field notes | To note observed or expressed beliefs, whether this affected observable engagement with the materials and whether this was associated with any particular content or delivery methods |
| Certainty: what are people’s levels of certainty about the extent of harm / benefit in working with industry | Pre- and post- survey question 4: certainty about evidence | We know that misinformation is provided by producers of harmful products. We wished to ensure that our briefing session increases certainty about the evidence that does exist. A ‘don’t know’ option was included to ensure participants could answer questions, whether they are familiar with the evidence or not. |
|  | Observation and field notes | To note observed or expressed certainty during the workshop and whether this was associated with any particular content or delivery methods |
| Skills: what skills do people report they have, what do they think they need | Pre- and post- survey questions 6b/c/d. | We want to know what skills people think they need and whether they have these skills. These are considered important pre-cursors to action. We want to know whether any of these answers change after the briefing session. |
| Optimism / self efficacy: How easy / hard do people feel it will be to take meaningful action? To what extent do people see it as their role to take action? How much control do they perceive themselves to have over the actions to take? | Pre- and post- survey questions 6a/b/d/e | We want to know whether people think it is important to take action on CDoH in general and whether they think it is part of their role. We want to know whether they have these skills and the confidence to take action. These are all considered important pre-cursors to action. We want to know whether any of these answers change after the briefing session. |
|  | Observation and field notes | To note observed or expressed self-efficacy during the workshop and whether this was associated with any particular content or delivery methods |
| What are people’s intentions and feelings about taking action? (including confidence in speaking to others about the issue) | Pre- and post- survey questions 6e (confidence) / 7 (action planned or underway) | We want to know whether people intend to take action and/or are already taking action. We want to know whether this changes before and after the workshop. |
|  | Observation and field notes | To note observed or expressed feelings and intentions about taking action during the workshop and whether this was associated with any particular content or delivery methods |
| What changes in working practices and working together have been reported as a result? | Self-report (3-month-follow up survey)  Report from colleagues in PH | To understand whether action has happened following the workshops and the extent to which participants report that the workshop may have contributed to subsequent action – as an indication of whether the workshops may help to generate action |

The surveys used with participants and the forms used for field notes and the topic guide for the reflective debriefs are available on request.

# Supplementary material 2

## Research sites

| Research site | Political control | Region | Urban / Rural | Type of authority |
| --- | --- | --- | --- | --- |
| A | Labour | Yorkshire & Humber | predominantly urban with major conurbation - has urban & rural mix | metropolitan district authority |
| B | Conservative | East Midlands | urban with significant rural, lower tier councils are categorised as a mix of predominantly rural, urban with significant rural, and predominantly urban - has a number of heavily built-up areas and large, sparsely populated rural areas – 27% of population live in rural areas | county council / upper tier authority |
| C | Labour | Yorkshire & Humber | predominantly urban with minor conurbation 17% ppn in rural areas | metropolitan district authority |
| D | Labour | North East | predominantly urban with major conurbation, has large rural area | metropolitan district authority |
| E | Liberal democrat | Yorkshire & Humber | predominantly urban with city and town | unitary authority |

## Workshop data

|  | Workshop 1 | Workshop 2 | Workshop 3 | Workshop 4 | Workshop 5 | Totals |
| --- | --- | --- | --- | --- | --- | --- |
| No. invited | 20 | 30 | 25 | 30 | 30 | 135 |
| Mix of invitees | core public health team and connected services: public health & leisure, communications, economic development and events, elected councillors | Wider public health team which includes the research, community safety, community wellness & leisure, libraries | Wider public health team which includes research and environmental health | Wider public health team, elected members, internal partners including trading standards, planning, licensing, economic regen, climate change, children’s, health & safety, comms, public protection | Public Health Senior Leadership team PH ELT (service mgr, ph lead, dep dir, dirs, strategic and operational managers) |  |
| No. planning to attend / accepted invite | 16 | 29 | 20 | 16 | 25 | 106 |
| No. consent forms received | 15 | 29 | 19 | 15 | 21 | 99 |
| No. attended on the day | 13 | 28 | 17 | 15 | 21 | 94 |
| Mix attended | 10 core public health, councillor, economic development and communications | Wider public health team which includes the research, community safety, community wellness & leisure, libraries | Wider public health team which includes research and environmental health | Wider public health team, internal partners including trading standards, planning, economic regen, climate change, children’s, health & safety, | Public Health Senior Leadership team PH ELT (service mgr, ph lead, dep dir, dirs, strategic and operational managers) |  |
| No. pre-workshop questionnaires completed | 12 | 26 | 15 | 12 | 21 | 86 |
| No. post-workshop questionnaires completed | 0 | 26 | 16 | 12 | 21 | 75 |
| No. follow up surveys completed | 5 | 8 | 13 | 4 | 9 | 39 |
| Additional notes | 1 councillor who was last-minute unable to attend was briefed by a local PH peer researcher using some of the materials after the workshop |  |  |  | 45 people were actually invited on the basis we would not have more than 30 attend as the time clashed with an internal training leadership programme so some wouldn't be able to attend |  |
| Planned length of workshop | 90 minutes | 90 minutes - advertised as 120 to allow 15 mins for arrival and pre-survey and 15 for post-survey and departure | 90 minutes - after experience in Workshop 1, asked people to arrive early and advised they would need to stay later however in reality not everyone did which squeezed the time for delivery | 90 minutes - advertised as 120 to allow 15 mins for arrival and pre-survey and 15 for post-survey and departure | 3hrs30mins including arrival & completion of survey, a break and completion of survey and chance to catch up at the end |  |
| Format | Face to face | Face to face | Face to face | Face to face | Face to face |  |
| Refreshments? | yes | yes | no | yes | yes |  |
| Room layout | cabaret | horseshoe | school room (desks for 2-3 facing fwd) | boardroom | cabaret |  |

# Supplementary material 3: Quantitative data

The time needed to complete the surveys was underestimated and this meant that workshop 1 ran over and participants had to leave before completing their post-workshop survey. The peer researchers from the local authority attempted to gather surveys after the workshop but were unable to. We therefore do not have data for the post-workshop survey for workshop 1. In all data where we are comparing pre and post, we report data excluding workshop 1, as this allows us to compare the same participants before and after.

|  | Pre-workshop | Post-workshop | Change |
| --- | --- | --- | --- |
| Knowledge | | | |
| Proportion of participants rating current knowledge of CDoH as 'a little' or 'none' | 55.41%  (n=74, 95%C.I. 44.08% to 66.73%) | 2.67%  (n=75, 95%C.I. -0.98% to 0.00%) | -52.74%  (95%C.I. -40.84% to -64.64%) |
| Expectations | | | |
| Proportion of participants stating the locally stated aims were met well or very well | n/a | 87.67%  (n=73, 95%C.I. 80.13% to 95.21%) | n/a |
| Skills, confidence, self-efficacy and optimism | | | |
| Proportion of participants agreeing or strongly agreeing that | | | |
| 'We should be taking action on the commercial determinants of health' | 89.04%  (n=73, 95%C.I. 81.88% to 96.21%) | 97.30%  (n=74, 95%C.I. 93.60% to 100.99%) | 8.26%  (95%C.I. 0.19% to 16.32%) |
| 'It is part of my role to take action on the commercial determinants of health' | 63.01%  (n=73, 95%C.I. 51.94% to 74.09%) | 87.67%  (n=73, 95%C.I. 80.13% to 95.21%) | 24.66%  (95%C.I. 11.26% to 38.06%) |
| 'I have these skills that I need to contribute to taking action on the commercial determinants of health' | 33.80%  (n=71, 95%C.I. 22.80% to 44.81%) | 54.79%  (n=73, 95%C.I. 43.38% to 66.21%) | 20.99%  (95%C.I. 5.14% to 36.85%) |
| 'I feel confident that I can contribute to taking action on the commercial determinants of health' | 45.21%  (n=73, 95%C.I. 33.79% to 56.62%) | 72.97%  (n=74, 95%C.I. 62.85% to 83.09%) | 27.77%  (95%C.I. 12.51% to 43.02%) |
| 'I feel optimistic that if we take action on the commercial determinants of health, this will result in positive outcomes' | 79.45%  (n=73, 95%C.I. 70.18% to 88.72%) | 80.82%  (n=73, 95%C.I. 71.79% to 89.85%) | 1.37%  (95%C.I. -11.57% to 14.31%) |
| Certainty about evidence | | | |
| Proportion of participants agreeing or strongly agreeing with the statement | | | |
| ‘There is evidence that some industries, sectors and companies advertise products that harm the population’s health’ | 93.24%  (n=74, 95%C.I. 87.52% to 98.96%) | 97.30%  (n=0, 95%C.I. 93.60% to 100.99%) | 4.05%  (95%C.I. -2.75% to 10.86%) |
| ‘There is evidence that some industries, sectors and companies have influenced policy in ways that harm the population’s health’ | 85.14%  (n=74, 95%C.I. 77.03% to 93.24%) | 98.65%  (n=74, 95%C.I. 96.02% to 101.28%) | 13.51%  (95%C.I. 4.99% to 22.04%) |

# Supplementary material 4: Theory of Change

## Theory of change

| **If we** | **Then we expect** | **And we expect this to lead to** |
| --- | --- | --- |
| Develop briefings with input from:   - Public Health - Academic experts - Feedback from internal partners who participate | - PH expectations to be met more closely - Briefings to be more clearly based on what has and hasn’t worked before - Internal partners’ expectations and needs to be met better | Briefings that cover appropriate content using effective delivery methods |
|  |  |  |
| Offer ‘briefings’ that include appropriate content using effective delivery methods including:   - evidence about the commercial determinants of health, how they operate, through which mechanisms and how they affect health - opportunities to reflect and practice skills   Additional implications / requirements identified during the research   - Need to ensure the facilitators are sufficiently skilled and trained and confident to run the workshops - Then need to ensure the local PH partners are as well - Then the workshops are about sharing that with their internal partners | People working with public health in local and regional systems to:   - Gain knowledge and understanding about the fundamental importance of CDoH - Have the opportunity to reflect on the need for change and how this fits with their prior beliefs and understanding and, if needed, integrate new paradigms into their current belief system - Be exposed to more evidence about the certainty of harm in the current system - Have the opportunity to practice skills (such as reviewing conflicts of interest or responding to media queries) - Identify what they are already doing that has a CDoH angle to it (note: this was added during the research process) | - their beliefs about the importance of this agenda to increase - their beliefs about the need for change to be more certain - their confidence in their ability to do something effective about it to increase - them to have or develop intention to take action   [additions during the research process]   - their understanding to increase and their ability to see what they're doing through a CDoH lens and understand some mechanisms through CDoH lens to increase - all the above to contribute to normalising talking and thinking about CDoH |
|  |  |  |
| Evaluate briefings | To understand what did and didn’t work for whom and in what circumstances | Ability to change the briefings to account for the evaluation |
| Iterate the briefings to take account of learning |  | To improve the effectiveness of the briefings |
| Deliver briefing sessions effectively and with enough people | To achieve the outcomes sought | This will contribute to the development of supportive environments for public health action on CDoH at local & regional level |

# References

Chinn, Deborah, and Catherine McCarthy. 2013. ‘All Aspects of Health Literacy Scale (AAHLS): Developing a Tool to Measure Functional, Communicative and Critical Health Literacy in Primary Healthcare Settings’. *Patient Education and Counseling* 90 (2). Elsevier: 247–253. doi:10.1016/J.PEC.2012.10.019.

Dahlgren, G, and Margaret Whitehead. 1993. *Tackling Inequalities in Health: What Can We Learn from What Has Been Tried?* Ditchley Park, Oxfordshire. London, King’s Fund (mimeo).

Elwell-Sutton T, Marshall L, Bibby J, Volmert A, and Health Foundation. 2019. *Reframing the Conversation on Social Determinants* . https://reader.health.org.uk/reframing-the-conversation-on-social-determinants/what-do-the-public-currently-think-about-health.

L’Hôte, Emilie, Marissa Fond, Andrew Volmert, and Frameworks Institute. 2018. ‘Seeing Upstream A FrameWorks Map the Gaps Report Commissioned by the Health Foundation’.
